# Crystal structure, Hirshfeld and electronic transition analysis of 2-[(1*H*-benzimidazol-1-yl)meth­yl]benzoic acid

**DOI:** 10.1107/S2056989021006435

**Published:** 2021-06-30

**Authors:** Arif Ali, Mohd Muslim, Saima Kamaal, Adeeba Ahmed, Musheer Ahmad, M. Shahid, Jamal A. Khan, Necmi Dege, Saleem Javed, Ashraf Mashrai

**Affiliations:** a Department of Applied Chemistry, Faculty of Engineering and Technology, ZHCET, Aligarh Muslim University, Aligarh 202002 (UP), India; b Functional Inorganic Materials Lab (FIML), Department of Chemistry, Aligarh, Muslim University, Aligarh 202002, India; c Ondokuz Mayis University, Faculty of Arts and Sciences, Department of, Physics,55139 Samsun, Turkey; d Department of Chemistry, Institute of H. Science, Dr. Bhimrao Ambedkar, University, Agra 282002, U. P., India; e Department of Pharmacy, University of Science and Technology, Ibb branch, Yemen

**Keywords:** crystal structure, benzimidazole, Tauc plot, Hirshfeld surface analysis

## Abstract

In the title compound, C_15_H_12_N_2_O_2_, the benzimidazole ring system is inclined to the benzene ring by 78.04 (10)°. The crystal structure features O—H⋯N and C—H⋯O hydrogen bonding and C—H⋯π and π–π inter­actions, which were investigated using Hirshfeld surface analysis.

## Abstract 2

In the title compound, the benzimidazole ring system is inclined to the the benzene ring by 78.04 (10)°. The crystal structure features O—H⋯N and C—H⋯O hydrogen bonding and C—H⋯π and π–π inter­actions.

## Chemical context   

Benzimidazole is a naturally ocurring compound, being present in vitamin B_12_ (Crofts *et al.*, 2014 ▸) and may also be synthesized from benzoic acid and *o*-phenyl­enedi­amine in presence of an excess of acid. Benzimidazole and its derivatives show biological activities such as anti­bacterial, anti­fungal (Yadav *et al.*, 2015 ▸), anti­microbial (Shruthi *et al.*, 2016 ▸), and anti­cancer (Kalalbandi *et al.*, 2015 ▸). Cyano­benzyl compounds are used as inter­mediates in the synthesis of species that possess significant pharmaceutical properties. Compounds having carb­oxy­lic acid as a functional group have shown chelating properties and thus have potential applications in the field of biology. Such groups are also helpful in building metal–organic frameworks that usually form supra­molecular networks due to extensive hydrogen bonding and weak inter­actions. For example, 4-[(1*H*-benzo[*d*]imidazol-1-yl)meth­yl]benzoic acid has been used to construct coordination polymers with different metal ions (Ahmad *et al.*, 2013 ▸). Herein, we report the title compound, 2-[(1*H*-benzimidazol-1-yl)meth­yl]benzoic acid, which was synthesized by a condensation reaction of benzimidazole and 2-(bromo­meth­yl) benzo­nitrile in aceto­nitrile followed by a hydrolysis process.
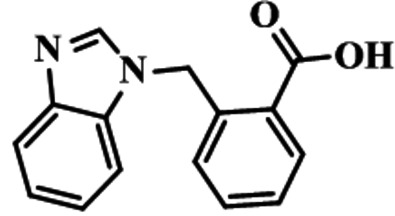


## Structural commentary   

**Figure 1:**
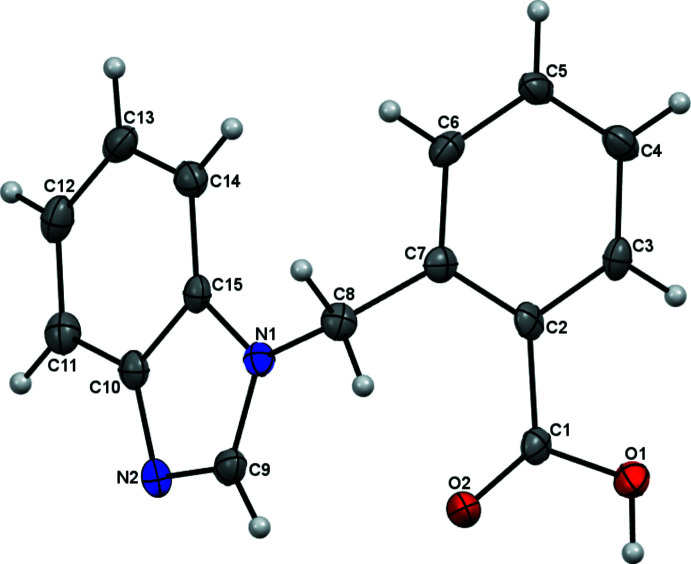
Asymmetric unit of title compound, with atom labelling and displacement ellipsoids are drawn at the 50% probability level.

The asymmetric unit of the title compound is illustrated in Fig. 1 ▸. The mol­ecule is non-planar with a dihedral angle of 78.04 (10) between the benzimidazole ring system and the benzene ring. The N1—C8—C7 angle is 113.31° and the C9—N1—C8—C7 torsion angle is −116.8 (2)°,. The C10—C15 bond length [1.408 (3) Å] is comparable to that in a similar benzimidazole derivative (Faizi *et al.*, 2017 ▸). The C—O bond lengths [C1—O1 = 1.319 (3) and C1—O2 = 1.216 (3) Å] are in the expected range (Kamaal *et al.*, 2019 ▸).

## Supra­molecular features   

**Figure 2:**
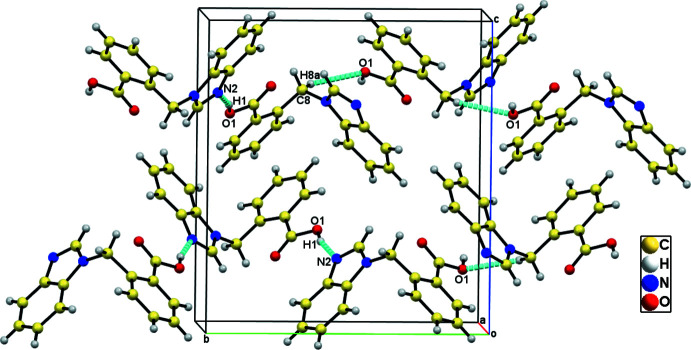
View of the crystal packing along the *a* axis, showing O—H⋯N and C—H⋯O hydrogen-bonding inter­actions forming a one-dimensional chain.

**Figure 3:**
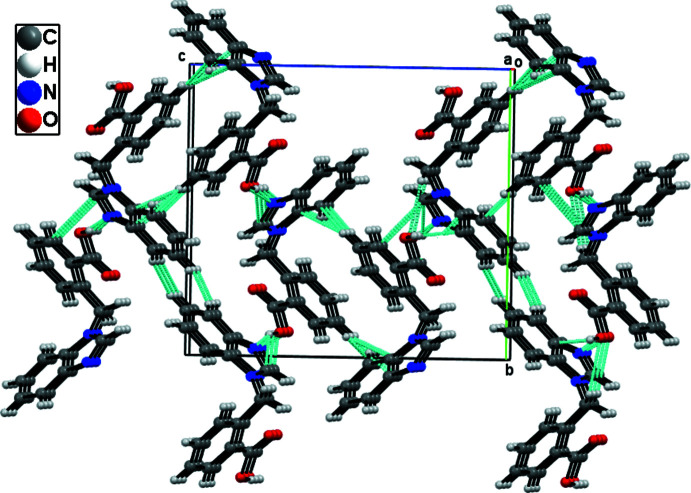
The hydrogen bonding and C—H⋯π and π–π inter­actions form zigzag chains, giving a supra­molecular structure along the *bc* plane.

In the crystal, the mol­ecules are connected *via* O—H⋯N and C—H⋯O hydrogen bonds (Table 1 ▸), forming a 1D framework along the *b-*axis direction (Fig. 2 ▸). C—H⋯π and π–π inter­actions [centroid–centroid distance = 3.6166 (15) Å] between the N1/N2/C9/C10/C15 and C2–C7 rings also occur, leading to the formation of the supra­molecular structure (Fig. 3 ▸).

## Database survey   

A search of the Cambridge Structural Database (CSD, Version 5.42, November 2020; Groom *et al.*, 2016 ▸) found five examples of similar compounds: bis­(penta­fluoro­phen­yl)-(μ-{1,1′-[1,2-phenyl­enebis(methyl­ene)]bis­(1*H*-benzimidazole)})digold(I) acetone solvate (WOPLIZ; Zheng *et al.*, 2019 ▸), 3,3′-[1,2-phenyl­enebis(methyl­ene)]bis­(1-ethyl­benzimidazolium) dibromide (LANHAL; Haque *et al.*, 2012 ▸), 2-[(1*H*-benzimidazol-1-yl)meth­yl]benzo­nitrile (JONYUJ; Akkoç *et al.*, 2017 ▸), 1-[(2-cyano­phen­yl)meth­yl]-3-[(2-methyl­phen­yl)meth­yl]-1*H*-benzimidazol-3-ium (JONZAQ; Akkoç *et al.*, 2017 ▸) and 1-(2-cyano­benz­yl)-3-methyl-1*H*-3,1-benzimidazol-3-ium bromide (MOCWAE; Ghdhayeb *et al.*, 2014 ▸).

## Hirshfeld surface analysis   

A Hirshfeld surface analysis was performed and the two-dimensional fingerprint plots generated (McKinnon *et al.*, 2007 ▸; Spackman & Jayatilaka *et al.*, 2009 ▸) using *CrystalExplorer17* (Turner *et al.*, 2017 ▸). The Hirshfeld surface mapped over *d*_norm_, colour-mapped from red (shorter distance than the sum of van der Waals radii) through white to blue (longer distance than the sum of the van der Waals radii). The principal weak inter­actions are clearly visible. The surface coverage corresponding to O—H⋯N and C—H⋯O inter­actions are 9% and 11.8%, respectively. The dark-red spot indicates significant hydrogen bonding.

**Figure 4:**
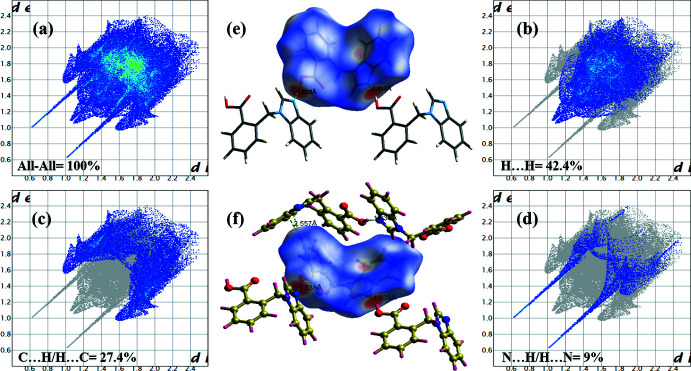
The Hirshfeld surface of the title compound mapped over *d*_norm_, in the range −0.722 to 1.183. (*a*) The overall two-dimensional finger plot of the title compound and those delineated into (*b*) H⋯H (42.4%), (*c*) C⋯H/ H⋯C (27.4%) and (*d*) N⋯H/H⋯N (9%) inter­actions, (*e*) significant hydrogen bonding and (*f*) extended supra­molecular form.

The two-dimensional finger plots are given in Fig. 4 ▸. The principal contributions to the overall surface are from H⋯H (42.4%, Fig. 4 ▸ *b*), C⋯H/H⋯C (27.4%, Fig. 4 ▸ *c*) and N⋯H/H⋯N 9% (Fig. 4 ▸ *d*) inter­actions. The contributions of inter­actions such as C⋯C 4.8% are negligible.

## Electronic transition analysis   

**Figure 5:**
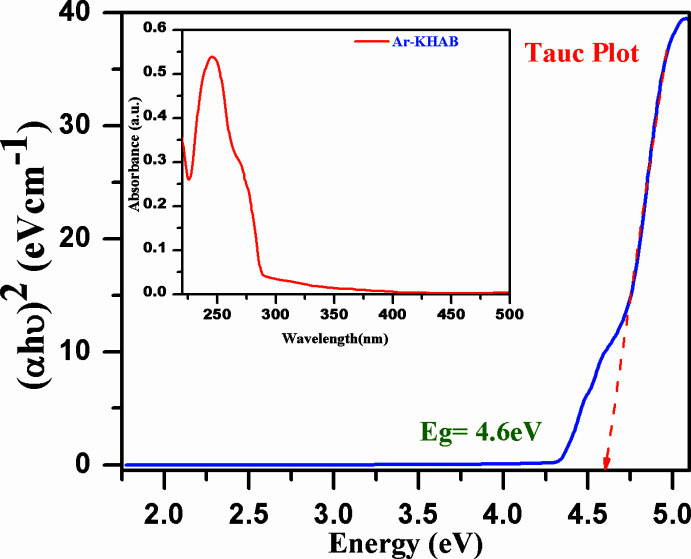
Energy band gap of the title mol­ecule by Tauc plot from absorption spectra.

Electro-conducting materials synthesized by conjugated organic compounds show promising electronic properties due to the availability of delocalized electrons, except for semiconducting materials such as TiO_2_, ZnO and other metal oxide nano-materials, which are electro-conducting in themselves (Odziomek *et al.*, 2017 ▸). The electronic properties of organic compounds depend on the electronic transition between the highest occupied mol­ecular orbital (HOMO) or valence band and lowest occupied mol­ecular orbital (LUMO) or conduction band. In a simple method, the energy band gap (*Eg*) of organic mol­ecule is determined by a Tauc plot from the absorption spectra (λ_max_ = 245 nm, in this case). The band gap energy, *Eg* = 4.6 eV, of the title compound is very large (Fig. 5 ▸). This large band gap arises due to high π-conjugation or polarization in the title mol­ecule system. The title mol­ecule could be useful for developing or enhancing the organic electronic properties of conducting materials such as metal–organic frameworks.

## Synthesis and crystallization   

In an equimolar ratio, benzimidazole (2 g, 16.9 mmol) and dry K_2_CO_3_ (4.66 g, 33.85 mmol) were mixed in a round-bottom flask in aceto­nitrile (MeCN, 60 ml) under an inert atmosphere. The mixture was then allowed to stirred for 60 min at 363 K then treated with 2-(bromo­meth­yl) benzo­nitrile (3.31 g, 16.9 mmol), and the resulting solution refluxed for 24 h. After completion of this step, the solution was allowed to cool to room temperature and the mixture was poured slowly onto ice–water (100 ml) under constant stirring. A greenish muddy crystalline precipitate was obtained and it was left to stand at 293 K for two days. After two days, a crystalline powder of 2-[(1*H*-benzo[*d*]imidazol-1-yl)meth­yl]benzo­nitrile was obtained (Ahmad *et al.*, 2013 ▸).

**Figure 6:**
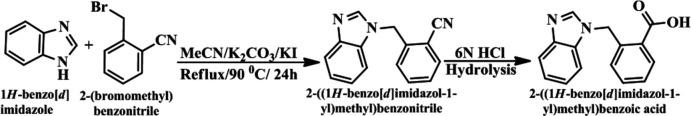
Reaction scheme.

The title compound was synthesized by hydrolysis of 2-[(1*H*-benzo[*d*]imidazol-1-yl)meth­yl]benzo­nitrile, 2 g being mixed with 20 equimolar of potassium hydroxide (6.86 g, 8.58 mmol) in water. The solution was refluxed at 373 K for 36 h, the resultant solution was then allowed to cool at room temperature and then poured onto ice–water, and after that acidified using 6 *N* HCl for protonation. The protonated solution was kept for slow evaporation. After two weeks, pale-yellow cubic crystals were obtained in good yield, which were suitable for data collection. The reaction scheme is shown in Fig. 6 ▸.

## Refinement   

Crystal data, data collection and structure refinement details are summarized in Table 2 ▸.

## Supplementary Material

Crystal structure: contains datablock(s) I. DOI: 10.1107/S2056989021006435/ex2044sup1.cif

Structure factors: contains datablock(s) I. DOI: 10.1107/S2056989021006435/ex2044Isup2.hkl

Supporting information file. DOI: 10.1107/S2056989021006435/ex2044Isup3.cml

CCDC reference: 2091351

Additional supporting information:  crystallographic information; 3D view; checkCIF report

## Figures and Tables

**Table 1:** Hydrogen-bond geometry (Å, °) *Cg*1, *Cg*2, *Cg*3 and *Cg4* are the centroids of the N1/N2/C9/C10/C15, C2–C7, C10–C15 and N1/N2/C9–15 rings, respectively.

*D*—H⋯*A*	*D*—H	H⋯*A*	*D*⋯*A*	*D*—H⋯*A*
O1—H1⋯N2^i^	0.88 (3)	1.73 (3)	2.592 (3)	164 (4)
C8—H8A⋯O1^ii^	0.99 (1)	2.62 (1)	3.374 (3)	133 (1)
C4—H4⋯*Cg*1^iii^	0.95 (1)	2.99 (1)	3.865 (3)	155 (1)
C4—H4⋯*Cg*3^iii^	0.95 (1)	2.51 (1)	3.408 (3)	157 (1)
C4—H4⋯*Cg*4^iii^	0.95 (1)	2.51 (1)	3.454 (3)	170 (1)
C5—H5⋯*Cg*2^iii^	0.95 (1)	2.76 (1)	3.554 (3)	142 (1)

Symmetry codes: (i) -x, y+{\script{1\over 2}}, -z+{\script{3\over 2}}; (ii) -x+1, y-{\script{1\over 2}}, -z+{\script{3\over 2}}; (iii) x+{\script{1\over 2}}, -y+{\script{3\over 2}}, -z+1.

**Table 2:** Experimental details

Crystal data	
Chemical formula	C_15_H_12_N_2_O_2_
*M*_r_	252.28
Crystal system, space group	Orthorhombic, *P*2_1_2_1_2_1_
Temperature (K)	100
*a*, *b*, *c* (Å)	6.5690 (8), 12.7956 (15), 14.1278 (16)
*V* (Å^3^)	1187.5 (2)
*Z*	4
Radiation type	Mo *K*α
μ (mm^−1^)	0.10
Crystal size (mm)	0.38 × 0.21 × 0.14

Data collection	
Diffractometer	Bruker APEXII CCD
Absorption correction	Multi-scan (*SADABS*; Bruker, 2014 ▸)
No. of measured, independent and observed [*I* ≥ 2u(*I*)] reflections	18798, 2095, 1759
*R*_int_	0.107
(sin θ/λ)_max_ (Å^−1^)	0.596

Refinement	
*R*[*F*^2^ > 2σ(*F* ^2^)], *wR*(*F* ^2^), *S*	0.044, 0.091, 1.09
No. of reflections	2095
No. of parameters	176
No. of restraints	1
H-atom treatment	All H-atom parameters refined
Δρ_max_, Δρ_min_ (e Å^−3^)	0.24, −0.28

Computer programs: *APEX2* and *SAINT* (Bruker, 2014 ▸), *olex2.solve* (Bourhis *et al.*, 2015 ▸), *olex2.refine* (Bourhis *et al.*, 2015 ▸) and *OLEX2* (Dolomanov *et al.*, 2009 ▸).

## supplementary crystallographic information

### Crystal data

**Table:**

C_15_H_12_N_2_O_2_	*D*_x_ = 1.411 Mg m^−^^3^
*M**_r_* = 252.28	Mo *K*α radiation, λ = 0.71073 Å
Orthorhombic, *P*2_1_2_1_2_1_	Cell parameters from 1209 reflections
*a* = 6.5690 (8) Å	θ = 2.9–22.1°
*b* = 12.7956 (15) Å	µ = 0.10 mm^−^^1^
*c* = 14.1278 (16) Å	*T* = 100 K
*V* = 1187.5 (2) Å^3^	Block, colourless
*Z* = 4	0.38 × 0.21 × 0.14 mm
*F*(000) = 528.253	

### Data collection

**Table:**

Bruker APEXII CCD diffractometer	1759 reflections with *I*≥ 2u(*I*)
Detector resolution: X-ray pixels mm^-1^	*R*_int_ = 0.107
φ and ω scans	θ_max_ = 25.1°, θ_min_ = 3.2°
Absorption correction: multi-scan (SADABS; Bruker, 2014)	*h* = −8→8
	*k* = −17→17
18798 measured reflections	*l* = −18→18
2095 independent reflections	

### Refinement

**Table:**

Refinement on *F*^2^	21 constraints
Least-squares matrix: full	Primary atom site location: iterative
*R*[*F*^2^ > 2σ(*F*^2^)] = 0.044	All H-atom parameters refined
*wR*(*F*^2^) = 0.091	*w* = 1/[σ^2^(*F*_o_^2^) + (0.0284*P*)^2^ + 0.3178*P*] where *P* = (*F*_o_^2^ + 2*F*_c_^2^)/3
*S* = 1.09	(Δ/σ)_max_ = 0.004
2095 reflections	Δρ_max_ = 0.24 e Å^−^^3^
176 parameters	Δρ_min_ = −0.28 e Å^−^^3^
1 restraint	

### Fractional atomic coordinates and isotropic or equivalent isotropic displacement parameters (Å^2^)

**Table:**

	*x*	*y*	*z*	*U*_iso_*/*U*_eq_	
C1	0.2861 (4)	0.82904 (18)	0.71973 (18)	0.0170 (6)	
C2	0.4789 (4)	0.81263 (19)	0.66504 (18)	0.0166 (6)	
C3	0.5267 (4)	0.88501 (19)	0.59451 (17)	0.0186 (6)	
H3	0.4333 (4)	0.93965 (19)	0.58083 (17)	0.0223 (8)*	
C4	0.7074 (4)	0.8790 (2)	0.54393 (18)	0.0213 (6)	
H4	0.7359 (4)	0.9282 (2)	0.49537 (18)	0.0256 (8)*	
C5	0.8447 (4)	0.80132 (19)	0.56462 (18)	0.0204 (7)	
H5	0.9705 (4)	0.79769 (19)	0.53166 (18)	0.0245 (8)*	
C6	0.7985 (4)	0.7280 (2)	0.63408 (18)	0.0196 (6)	
H6	0.8941 (4)	0.6743 (2)	0.64778 (18)	0.0235 (7)*	
C7	0.6164 (4)	0.7311 (2)	0.68391 (17)	0.0163 (6)	
C8	0.5804 (4)	0.6465 (2)	0.75638 (17)	0.0179 (6)	
H8a	0.7114 (4)	0.6115 (2)	0.77035 (17)	0.0215 (7)*	
H8b	0.5313 (4)	0.6790 (2)	0.81572 (17)	0.0215 (7)*	
C9	0.2503 (4)	0.54778 (19)	0.76721 (18)	0.0197 (6)	
H9	0.2023 (4)	0.58470 (19)	0.82111 (18)	0.0236 (7)*	
C10	0.2677 (4)	0.43913 (19)	0.65142 (17)	0.0176 (6)	
C11	0.2311 (4)	0.3622 (2)	0.58413 (19)	0.0229 (6)	
H11	0.1089 (4)	0.3224 (2)	0.58480 (19)	0.0274 (8)*	
C12	0.3793 (4)	0.3456 (2)	0.51608 (19)	0.0248 (7)	
H12	0.3580 (4)	0.2940 (2)	0.46874 (19)	0.0297 (8)*	
C13	0.5601 (4)	0.40366 (19)	0.51595 (18)	0.0223 (7)	
H13	0.6590 (4)	0.39013 (19)	0.46844 (18)	0.0267 (8)*	
C14	0.5993 (4)	0.4801 (2)	0.58275 (18)	0.0197 (6)	
H14	0.7226 (4)	0.5188 (2)	0.58240 (18)	0.0236 (8)*	
C15	0.4494 (4)	0.49744 (19)	0.65048 (17)	0.0156 (6)	
N1	0.4321 (3)	0.56758 (15)	0.72553 (14)	0.0158 (5)	
N2	0.1467 (3)	0.47270 (16)	0.72598 (15)	0.0195 (5)	
O1	0.1923 (3)	0.91615 (15)	0.69535 (13)	0.0228 (5)	
O2	0.2246 (3)	0.77045 (13)	0.78116 (12)	0.0227 (4)	
H1	0.078 (4)	0.924 (3)	0.727 (2)	0.077 (13)*	

### Atomic displacement parameters (Å^2^)

**Table:**

	*U*^11^	*U*^22^	*U*^33^	*U*^12^	*U*^13^	*U*^23^
C1	0.0165 (14)	0.0131 (13)	0.0214 (14)	−0.0029 (12)	−0.0021 (12)	−0.0043 (12)
C2	0.0186 (14)	0.0126 (13)	0.0184 (14)	−0.0064 (11)	−0.0017 (12)	−0.0040 (11)
C3	0.0246 (16)	0.0118 (14)	0.0193 (14)	0.0002 (12)	−0.0039 (12)	−0.0027 (11)
C4	0.0257 (16)	0.0207 (14)	0.0175 (14)	−0.0068 (13)	0.0054 (13)	−0.0014 (12)
C5	0.0217 (15)	0.0172 (14)	0.0223 (16)	−0.0060 (12)	0.0068 (12)	−0.0042 (12)
C6	0.0187 (14)	0.0166 (13)	0.0235 (15)	0.0024 (12)	−0.0007 (12)	−0.0050 (12)
C7	0.0166 (13)	0.0158 (14)	0.0164 (13)	−0.0037 (12)	−0.0020 (11)	−0.0056 (11)
C8	0.0166 (13)	0.0181 (14)	0.0190 (14)	−0.0010 (12)	−0.0033 (12)	−0.0009 (12)
C9	0.0241 (15)	0.0163 (14)	0.0186 (14)	0.0038 (12)	0.0022 (13)	0.0027 (12)
C10	0.0210 (14)	0.0149 (13)	0.0170 (13)	0.0010 (12)	−0.0025 (12)	0.0032 (11)
C11	0.0235 (15)	0.0183 (14)	0.0268 (15)	−0.0006 (13)	−0.0034 (13)	0.0007 (12)
C12	0.0339 (17)	0.0159 (14)	0.0244 (16)	0.0026 (14)	−0.0028 (14)	−0.0030 (13)
C13	0.0295 (17)	0.0185 (15)	0.0188 (14)	0.0077 (12)	0.0031 (13)	0.0007 (12)
C14	0.0201 (15)	0.0178 (14)	0.0212 (14)	−0.0002 (12)	0.0010 (12)	0.0031 (12)
C15	0.0198 (15)	0.0117 (13)	0.0153 (13)	0.0008 (11)	−0.0027 (12)	0.0017 (11)
N1	0.0160 (12)	0.0144 (11)	0.0170 (11)	−0.0010 (9)	−0.0006 (10)	0.0000 (10)
N2	0.0222 (12)	0.0136 (12)	0.0226 (12)	−0.0009 (10)	0.0014 (11)	0.0032 (10)
O1	0.0170 (11)	0.0199 (10)	0.0316 (11)	0.0030 (9)	0.0012 (9)	0.0042 (9)
O2	0.0250 (10)	0.0171 (9)	0.0259 (11)	0.0012 (8)	0.0075 (9)	0.0031 (9)

### Geometric parameters (Å, º)

**Table:**

C1—C2	1.499 (3)	C9—H9	0.950 (4)
C1—O1	1.319 (3)	C9—N1	1.356 (3)
C1—O2	1.216 (3)	C9—N2	1.313 (3)
C2—C3	1.396 (3)	C10—C11	1.390 (3)
C2—C7	1.405 (3)	C10—C15	1.408 (3)
C3—H3	0.950 (4)	C10—N2	1.388 (3)
C3—C4	1.388 (3)	C11—H11	0.9501 (4)
C4—H4	0.950 (4)	C11—C12	1.385 (4)
C4—C5	1.373 (4)	C12—H12	0.950 (4)
C5—H5	0.950 (4)	C12—C13	1.401 (4)
C5—C6	1.391 (4)	C13—H13	0.950 (4)
C6—H6	0.951 (4)	C13—C14	1.383 (3)
C6—C7	1.389 (4)	C14—H14	0.949 (4)
C7—C8	1.508 (3)	C14—C15	1.391 (4)
C8—H8a	0.990 (4)	C15—N1	1.394 (3)
C8—H8b	0.990 (3)	O1—H1	0.878 (18)
C8—N1	1.469 (3)		
			
O1—C1—C2	112.3 (2)	N1—C9—H9	123.20 (14)
O2—C1—C2	124.2 (2)	N2—C9—H9	123.20 (15)
O2—C1—O1	123.5 (2)	N2—C9—N1	113.6 (2)
C3—C2—C1	117.7 (2)	C15—C10—C11	121.1 (2)
C7—C2—C1	123.3 (2)	N2—C10—C11	129.7 (2)
C7—C2—C3	118.9 (2)	N2—C10—C15	109.2 (2)
H3—C3—C2	119.23 (15)	H11—C11—C10	121.25 (15)
C4—C3—C2	121.5 (2)	C12—C11—C10	117.5 (3)
C4—C3—H3	119.23 (15)	C12—C11—H11	121.25 (16)
H4—C4—C3	120.26 (15)	H12—C12—C11	119.48 (16)
C5—C4—C3	119.5 (2)	C13—C12—C11	121.0 (2)
C5—C4—H4	120.26 (15)	C13—C12—H12	119.48 (15)
H5—C5—C4	120.16 (15)	H13—C13—C12	118.94 (15)
C6—C5—C4	119.7 (2)	C14—C13—C12	122.1 (2)
C6—C5—H5	120.16 (16)	C14—C13—H13	118.94 (16)
H6—C6—C5	119.13 (16)	H14—C14—C13	121.59 (16)
C7—C6—C5	121.7 (2)	C15—C14—C13	116.8 (3)
C7—C6—H6	119.13 (16)	C15—C14—H14	121.59 (16)
C6—C7—C2	118.6 (2)	C14—C15—C10	121.5 (2)
C8—C7—C2	124.1 (2)	N1—C15—C10	105.3 (2)
C8—C7—C6	117.3 (2)	N1—C15—C14	133.2 (2)
H8a—C8—C7	108.91 (14)	C9—N1—C8	125.7 (2)
H8b—C8—C7	108.91 (13)	C15—N1—C8	127.9 (2)
H8b—C8—H8a	107.7 (3)	C15—N1—C9	106.4 (2)
N1—C8—C7	113.31 (19)	C10—N2—C9	105.4 (2)
N1—C8—H8a	108.91 (12)	H1—O1—C1	111 (2)
N1—C8—H8b	108.91 (12)		
			
C1—C2—C3—C4	176.5 (2)	C8—N1—C9—N2	−179.6 (2)
C1—C2—C7—C6	−174.8 (2)	C8—N1—C15—C10	179.7 (3)
C1—C2—C7—C8	4.3 (3)	C8—N1—C15—C14	−1.3 (3)
C2—C3—C4—C5	−1.2 (3)	C9—N1—C15—C10	1.0 (2)
C2—C7—C6—C5	−1.9 (3)	C9—N1—C15—C14	179.9 (2)
C2—C7—C8—N1	75.2 (3)	C9—N2—C10—C11	−179.2 (2)
C3—C4—C5—C6	1.8 (3)	C9—N2—C10—C15	0.4 (2)
C4—C5—C6—C7	−0.2 (3)	C10—C11—C12—C13	0.6 (3)
C5—C6—C7—C8	178.9 (2)	C10—C15—C14—C13	0.7 (3)
C6—C7—C8—N1	−105.6 (2)	C11—C12—C13—C14	−0.3 (3)
C7—C8—N1—C9	−116.8 (2)	C12—C13—C14—C15	−0.4 (3)
C7—C8—N1—C15	64.7 (3)	C13—C14—C15—N1	−178.1 (2)

### Hydrogen-bond geometry (Å, º)

*Cg*1, *Cg*2, *Cg*3 and *Cg4* are the centroids of the N1/N2/C9/C10/C15, C2–C7, C10–C15 and N1/N2/C9–15 rings, respectively.

**Table:**

*D*—H···*A*	*D*—H	H···*A*	*D*···*A*	*D*—H···*A*
O1—H1···N2^i^	0.88 (3)	1.73 (3)	2.592 (3)	164 (4)
C8—H8A···O1^ii^	0.99 (1)	2.62 (1)	3.374 (3)	133 (1)
C3—H3···O1	0.95 (1)	2.28 (1)	2.648 (3)	102 (1)
C8—H8B···O2	0.99 (1)	2.38 (1)	2.846 (3)	108 (1)
C9—H9···O2	0.95 (1)	2.45 (1)	2.861 (3)	106 (1)
C4—H4···*Cg*1^iii^	0.95 (1)	2.99 (1)	3.865 (3)	155 (1)
C4—H4···*Cg*3^iii^	0.95 (1)	2.51 (1)	3.408 (3)	157 (1)
C4—H4···*Cg*4^iii^	0.95 (1)	2.51 (1)	3.454 (3)	170 (1)
C5—H5···*Cg*2^iii^	0.95 (1)	2.76 (1)	3.554 (3)	142 (1)

Symmetry codes: (i) −*x*, *y*+1/2, −*z*+3/2; (ii) −*x*+1, *y*−1/2, −*z*+3/2; (iii) *x*+1/2, −*y*+3/2, −*z*+1.
